# Prediction and Identification of T Cell Epitopes in the H5N1 Influenza Virus Nucleoprotein in Chicken

**DOI:** 10.1371/journal.pone.0039344

**Published:** 2012-06-20

**Authors:** Yanxia Hou, Yingying Guo, Chunyan Wu, Nan Shen, Yongping Jiang, Jingfei Wang

**Affiliations:** 1 Centre for Animal Infectious Disease Diagnosis and Technical Services and State Key Laboratory of Veterinary Biotechnology, Harbin Veterinary Research Institute, Chinese Academy of Agricultural Sciences, Harbin, Heilongjiang, People’s Republic of China; 2 Animal Influenza Laboratory of the Ministry of Agriculture and State Key Laboratory of Veterinary Biotechnology, Harbin Veterinary Research Institute, Chinese Academy of Agricultural Sciences, Harbin, Heilongjiang, People’s Republic of China; Russian Academy of Sciences, Institute for Biological Instrumentation, Russian Federation

## Abstract

T cell epitopes can be used for the accurate monitoring of avian influenza virus (AIV) immune responses and the rational design of vaccines. No T cell epitopes have been previously identified in the H5N1 AIV virus nucleoprotein (NP) in chickens. For the first time, this study used homology modelling techniques to construct three-dimensional structures of the peptide-binding domains of chicken MHC class Ι molecules for four commonly encountered unique haplotypes, i.e., B4, B12, B15, and B19. H5N1 AIV NP was computationally parsed into octapeptides or nonapeptides according to the peptide-binding motifs of MHC class I molecules of the B4, B12, B15 and B19 haplotypes. Seventy-five peptide sequences were modelled and their MHC class I molecule-binding abilities were analysed by molecular docking. Twenty-five peptides (Ten for B4, six for B12, two for B15, and seven for B19) were predicted to be potential T cell epitopes in chicken. Nine of these peptides and one unrelated peptide were manually synthesized and their T cell responses were tested *in vitro*. Spleen lymphocytes were collected from SPF chickens that had been immunised with a NP-expression plasmid, pCAGGS-NP, and they were stimulated using the synthesized peptides. The secretion of chicken IFN-γ and the proliferation of CD8^+^ T cells were tested using an ELISA kit and flow cytometry, respectively. The significant secretion of chicken IFN-γ and proliferation of CD8^+^ T lymphocytes increased by 13.7% and 11.9% were monitored in cells stimulated with peptides NP_89–97_ and NP_198–206_, respectively. The results indicate that peptides NP_89–97_ (PKKTGGPIY) and NP_198–206_ (KRGINDRNF) are NP T cell epitopes in chicken of certain haplotypes. The method used in this investigation is applicable to predicting T cell epitopes for other antigens in chicken, while this study also extends our understanding of the mechanisms of the immune response to AIV in chicken.

## Introduction

The introduction into the human population of animal-derived influenza A viruses with a novel haemagglutinin (HA), or a novel HA and neuraminidase (NA), and their subsequent spread could result in global influenza pandemics [1]. Since 2003, the highly pathogenic H5N1 avian influenza virus (AIV) has caused numerous cases of severe disease and death in humans [2]. An influenza pandemic could ensue if this virus developed the capacity to spread easily among humans [3]–[5]. Migratory birds constitute the natural reservoir for AIVs, but chickens may play a key role in the transmission to humans [6]. Epitopes can be used for accurately monitoring immune responses to AIV and for the rational design of protective vaccines. However, only two epitopes from the H5N1 avian influenza A/Vietnam/1194/2004 virus are included in the Immune Epitope Database and Analysis Resources (IEDB). The majority of T cell and B cell epitopes have been identified in mouse, human, or rabbit hosts. Few epitopes have been described in chicken [7].

The structural basis of peptide binding to mammalian major histocompatibility complex (MHC) class I molecules is well understood [8]. The peptide-binding groove is formed by the α1 and α2 domains. Each domain contributes four strands to an eight -stranded anti-parallel β-sheet. Two long interrupted helices, one from each domain, pack against the side of this sheet in an orientation directed away from the cell membrane. There is a series of pockets (A–F) along the peptide-binding groove where highly polymorphic amino acids mediate recognition via haplotype-specific associations with antigens and T cell receptors. However, highly conserved residues are found at both ends of the peptide-binding groove that form a network of hydrogen bonds, which directly interact with hydrogen bonds at the peptide’s N-terminus and C-terminus [9]–[11]. Peptides that bind to MHC class I molecules are usually octamers or nonamers, where only one or a few residues can interact with polymorphic residues in the groove. These residues are known as anchor residues when they are found in an anchoring position [12]–[15]. Several public databases and prediction services are available for MHC molecular ligands and peptide motifs, including SYFPEITHI and RANKPEP [16], [17].

Unlike mammalian studies, the majority of investigations of MHC class I molecules in chicken remain limited to primary sequences and the structure of MHC class I molecule from the B21 haplotype was solved only recently [18], [19]. The chicken MHC B system is located on chicken Chromosome 16 (Chr16) and is composed of tightly linked polymorphic regions: BF (class I) and BL (class IIβ) and a large family of polymorphic Ig-superfamily (IgSF) genes called BG–the latter sharing sequence similarities with mammalian MHC butyrophilin, myelin oligodendrocyte glycoprotein, and TRIM genes [20], [21]. The MHC B–F molecules are structurally and functionally similar to mammalian MHC class I molecules. The two class I genes are known as BF1 and BF2, although the BF2 gene is mainly expressed. The MHC class I (B–F) molecules present antigen peptides to the CD8^+^ T lymphocytes, which have a central role in the immune system. The MHC class I molecules of different haplotypes have specific peptide-binding tropisms, and B–F-associated peptide-binding motifs have been determined for several haplotypes, including B4, B12, B15, and B19 [22]. Thirteen peptides derived from the v-src gene of the Rous sarcoma virus (RSV) Prague strain were predicted to fit the peptide-binding motif of the MHC class I molecules of B12 haplotype and all 13 synthetic peptides actually bound to the BF12 class I molecule in subsequent binding tests [23]. This demonstrates that peptide-binding motifs can be used to predict the antigen peptides presented by chicken MHC class I molecules.

However, no T cell epitope of H5N1 AIV NP has yet been identified in the chicken [7], while no information is available on the structure of chicken MHC class Ι molecules belonging to the B4, B12, B15, and B19 haplotypes. For the first time, the current study reports the homology modelling structures of the peptide-binding domains of chicken MHC class Ι molecules for the B4, B12, B15, and B19 haplotypes. Potential T cell epitopes were predicted by molecular docking of 25 peptides in the H5N1 AIV NP in chicken. NP_89–97_ and NP_198–206_ were shown to be T cell epitopes of H5 AIV NP by analysing the CD8^+^ T cell proliferation and the interferon (IFN-γ) expression. To our knowledge, this is the first report to describe the structure of chicken MHC class Ι molecules from the B4, B12, B15 and B19 haplotypes and the T cell epitopes of H5N1 AIV NP in chicken.

## Materials and Methods

### Hardware and Software

All computations were conducted using the Discovery Studio 2.5 (DS2.5) program developed by Accelrys Software Inc and the SYBYL 8.1 program developed by Tripos Inc on an SGI Fuel workstation running Red Hat Enterprise 5.3 and a Dell server running the Red Hat Enterprise 5.2 Linux operating system.

### Animals

SPF chickens were housed in HEPA-filtered isolators.

### Ethics Statement

Chicken lymphocytes collected from immunized chickens, which were approved by Harbin Veterinary Research Institute, Chinese Academy of Agricultural Sciences and performed in accordance with animal ethics guidelines and approved protocols. The Animal Ethics Committee approval number is Heilongjiang-SYXK-2006-032.

### NP Expression Plasmid pCAGGS-NP

DNA vaccine pCAGGS-NP was constructed and assessed according to Ma and Jiang [24], [25]. The synthetic gene for NP of the isolate A/Goose/Gongdong/1/96 (H5N1) with codons optimized for chicken usage was synthesized by PCR assembly of long single-strand DNA templates (100 bases in length) (the oligonucleotide sequences are available upon request). The synthetic optiNP was cloned into the plasmid vector pCAGGS under the control of the chicken β-actin promoter. The plasmid was named pCAGG-NP. The expression of the NP protein from the plasmid was confirmed by indirect immunofluorescence assay and Western blotting of plasmid transfected CEF cells.

### Peptides

Nine of the predicted peptides (Table 1) were selected to test their T cell responses in vitro. They were synthesized to >90% purity by the GenScript Corporation (Nanjing, China): NP_7–14_ (KRSYEQME), NP_127–134_ (EDATAGLT), NP_477–484_ (PSFDMSNE), NP_25–33_ (IRASVGRMV), NP_190–197_ (VMELIRMI), NP_76–83_ (NKYLEEHP), NP_89–97_ (PKKTGGPIY), NP_198–206_ (KRGINDRNF), NP_64–71_ (ERMVLSAF), and the unrelated peptide N_71–78_ (WRRQARYK) [26].

**Table 1:** Predicted T cell epitopes in H5N1 AIV NP for four chicken haplotypes.

Chicken MHC haplotype	Motif	Position	Sequence	Score	Binding energy (Kcal mol^–1^)
B4	**xxxxExxE**	**7–14**	**KRSYEQME**	**11.73**	**−134.66178**
	xExxExxx	10–17	YEQMETGG	12.63	−124.61178
	xxxxxxxE	66–73	MVLSAFDE	16.23	−127.08578
	xxxxDxxx	124–131	NNGEDATA	12.46	−131.64478
	xExxxxxx	126–133	GEDATAGL	11.11	−132.12278
	**xDxxxxxx**	**127–134**	**EDATAGLT**	**8.64**	**−135.70778**
	xExxDxxx	251–258	AEIEDLIF	10.37	−118.38878
	xxxxxxxE	362–369	GVQIASNE	15.19	−131.87878
	xExxxxxx	453–460	PEDVSFQG	12.97	−122.84178
	**xxxxxxxE**	**477–484**	**PSFDMSNE**	**9.8**	**−139.58678**
B12	**xxxxVxxxV**	**25–33**	**IRASVGRMV**	**18.07**	**−199.38597**
	xxxxVxxx	29–36	VGRMVGGI	12.72	−198.51497
	xxxxVxxV	179–186	AGAAVKGV	11.42	−196.12897
	xxxxVxxx	186–192	VGTMVMEL	10.15	−191.12797
	**xxxxIxxx**	**190–197**	**VMELIRMI**	**14.87**	**−204.01297**
	xxxxIxxx	249–256	GNAEIEDL	13.3	−195.53997
B15	**xRxxxxxx**	**64–71**	**ERMVLSAF**	**12.76**	**−184.79194**
	xxxxxxxY	90–97	KKTGGPIY	11.27	−194.13594
B19	xRxxxxxF	64–71	ERMVLSAF	14.85	−197.85904
	**xxxxxxxP**	**76–83**	**NKYLEEHP**	**12.7**	**−205.59404**
	**xxxxxxxxY**	**89–97**	**PKKTGGPIY**	**14.36**	**−195.93004**
	xxxxxxxxL	102–110	GKWVRELIL	13.26	−198.0004
	**xRxxxxxxF**	**198–206**	**KRGINDRNF**	**15.27**	**−203.86004**
	xRxxxxxxP	445–453	IRMMESSRP	17.23	−184.64204
	xRxxxxxF	451–458	SRPEDVSF	11.06	−193.96604

Note: The peptides in bold font were synthesized to test their T cell responses in vitro.

### Experimental Design

Amino sequence of H5N1 AIV(GD/1/96) NP was parsed into octamers or nonamers according to the peptide-binding motifs of MHC class I molecules belonging to the B4, B12, B15, and B19 haplotypes. The 3D structures of those peptides and the MHC class I molecules were predicted using homology modelling method and then the binding affinity between each peptide and MHC class I molecule was analysed using molecular docking. Those peptides, which have correct binding conformation and high binding affinity, were predicted to be potential T cell epitopes in chicken. Predicted peptides 9 of 25 were synthesized and used to stimulate splenic lymphocytes collected from NP DNA vaccine-immunized SPF chickens. MHC class I molecule-restricted potential T-cell epitopes were confirmed by detecting of CD8^+^ T cell proliferation and interferon (IFN-γ) expression.

### Homology Modelling and Structure Analysis

The amino acid sequences of chicken MHC class I molecules belonging to the B4, B12, B15, and B19 haplotypes were obtained from GenBank (B4, GenBankID: CAK54654.1; B12, GenBankID: BAG69386.1; B15, GenBankID: BAG69413.1; and B19, GenBankID: BAG69441.1). BLASTP was performed to search for homologous proteins in the Protein Data Bank (PDB) using the BlOSUM62 Scoring Matrix optimized with a gap penalty of 11 and a gap extension penalty of 1. The MODELER [27] program was then executed in DS2.5 to construct the 3D structures of chicken MHC class I molecules for the four haplotypes. The quality of the 3D models was evaluated with a Ramachandran Plot using the PROCHECK [28] program and the Verify Protein (Profiles-3D) program [29] in DS2.5.

To improve the quality of the models, unsatisfied loop regions in each model were refined using the loop-refinement protocol based on the MODELER energy in DS 2.5. All structures were then minimized using the CHARMm force field [30] and an explicit solvent model (TIP3P water) with a steepest descent method for 8000 steps and a conjugated gradient minimization for a further 8000 steps.

The cavity depth, lipophilic potential (LP), flexibility (FX), and electrostatic potential (EP) of the four structures was analysed using the MOLCAD program in SYBYL8.1. During this process, the Gasteiger-Hückel charges were assigned to all atoms, while surface maps were generated and visualized using SYBYL8.1.

### Preparation of the Peptides

The NP protein sequence of H5N1 isolate A/Goose/Gongdong/1/96 (H5N1) was downloaded from the UniProt database (UniProtID: NCAP_I96A0) and automatically parsed as octapeptides or nonapeptides using a computer program, which was developed in our laboratory, based on the peptide-binding motifs of chicken MHC class I molecules belonging to the B4, B12, B15, and B19 haplotypes [22]. The motifs were as follows: B4: x-(D or E)-x-x-(D or E)-x-x-E; B12: x-x-x-x-(V or I)-x-x-V and x-x-x-x-(V or I)-x-x-x(V); B15: (K or R)-R-x-x-x-x-x-Y and (K or )-R-x-x-x-x-x-x-Y; B19: x-R-x-x-x-x-x-(Y, P, L, F) and x-R-x-x-x-x-x-x-(Y, P, L, F). The structures of the octapeptides and nonapeptides were modelled using DS2.5. In total, seventy-five peptide structures were prepared for docking analysis.

### Molecular Docking

Surflex-Dock [31] is used to dock ligands into a protein-binding site and it is particularly successful at eliminating false positive results [32] while offering unparalleled enrichment in virtual high-throughput screening combined with state-of-the-art speed, accuracy, and usability [33], [34]. To test the feasibility of using Surflex-Dock for docking MHC molecules and peptides, 50 peptide-MHC complexes retrieved from the PDB database were separated and re-docked using the Surflex-Dock program. The RMSD was calculated for each modelled pair and crystal structure pair to evaluate the docking program.

Surflex-Dock was used to dock the octapeptides and nonapeptides to their corresponding MHC class I molecule receptors, where the parameters of the threshold and bloat values of the program were optimized to 0.51 and 1. The interaction modes and binding energies of the docked complexes were analysed using DS2.5. Peptides with a higher docking score and rational conformation were predicted as candidate T cell epitopes for each haplotype.

### Immunization of SPF Chickens with Plasmid pCAGGS-NP

For the DNA vaccine immunizations, 13 three-week-old SPF chickens were separated into two groups, eight were immunized twice with 100 µg of pCAGGS-NP in their leg muscle at three-week intervals, while five chickens were injected with the same volume of PBS as controls. Sera were collected weekly for detecting of NP antibody.

### Detection of NP Antibody

Serum antibodies to H5N1 AIV NP were detected using an indirect ELISA method as described by [24], which used prokaryotically-expressed NP as the antigen. The testing steps as follows, after washing of the plates, 50 µl of the test serum mixed in the test wells with 50 µl of antigen diluted 1∶10 in ELISA buffer. After incubation at 37°C for 1 h, 100 µl horseradish peroxidase conjugate, diluted 1∶1000 in ELISA buffer, was added to each well and plates were further incubated at 37°C for 1 h. After two washing steps, 100 µl of TMB substrate was added and incubated at room temperature for 10 min. The reaction was stopped by adding 100 µl H_2_SO_4_ 2 M. The extinctions were measured at 490 nm with a micro ELISA reader (Bio-Rad).

### Preparation of Splenic Lymphocytes

To prepare splenic lymphocytes, all eight pCAGGS-NP immunized chickens were killed by cardiac puncture blood collection. Sterile spleens were collected and meshed through a sieve screen using a syringe plunger to obtain a single-cell suspension in tissue culture medium (RPMI 1640, Gibco BRL NY, USA). Cell suspensions were overlaid onto Histopaque 1077 density gradient medium and centrifuged at 1800 rpm for 20 min at 18°C. Lymphocytes were collected from the interface and washed three times in RPMI, before cells were counted using a trypan blue dye exclusion assay.

### CD8^+^ T Lymphocyte Proliferation Assay

Splenic lymphocytes collected from the 8 immunized chickens were stained with 5 µM CFSE (Invitrogen) in pre-warmed PBS for 10 min, washed three times, and suspended in RPMI 1640 containing 2 mM L-glutamine, 100 IU mL^–1^ penicillin, 100 IU mL^–1^ streptomycin, and 10% foetal bovine serum (R10 medium). Cells were plated at 10^6^ well^–1^ in 24-well plates with RPMI 1640 medium, before stimulation for 5d with 100 µg mL^–1^ of the nine peptides generated from NP and the unrelated peptide N_71–78_. Cells were then washed with PBS and stained using anti-CD8-PE before flow cytometric analysis, which was performed on Cytomics FC500 MCL(Beckman) and analysed with its embedded software CXP. Two repeats were performed simultaneously for each peptide during the flow cytometric analysis.

### Detection of Chicken IFN-γ

Splenic lymphocytes collected form the 8 vaccinated chickens were plated at 10^6^ well^–1^ in 24-well plates with RPMI 1640 medium, before stimulation for 48 h using 100 µg mL^–1^ of the nine peptides generated from NP and the unrelated peptide N_71–78_. Cells were then collected and centrifuged at 1000 rpm for 5 min. The cell culture supernatants were then analysed to determine the chicken IFN-γ using chicken IFN-γ CytoSets™ (Invitrogen), according to the manufacturer’s instructions [35]. The extinctions were measured at 450 nm with a micro ELISA reader (Bio-Rad). For each peptide, two copies of splenic lymphocytes from one chicken were treated and analysed simultaneously.

### Statistical Analysis

Results were expressed as mean ± S.E.M. for two replicates. Statistical analyses were performed using SPSS 16.0 for Windows. Significant differences (*P*<0.05) between means were tested by one-way ANOVA, followed by Tukey’s Honestly Significant Difference test.

## Results

### Quality of the Models

**Figure 1:**
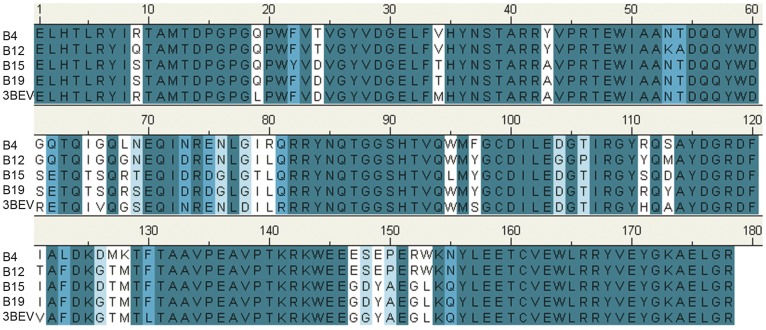
Multiple sequence alignment of the α1 and α2 domains of chicken MHC class I molecules (B4, B12, B15, and B19 haplotypes) with the template B21 haplotype (PDB:3BEV).

The four haplotypes selected in this study belonged to 11 commonly encountered unique haplotypes (B2, B4, B5, B6, B7, B12, B13, B14, B15, B19, and B21) and the motifs of their binding peptides were previously reported [22], [23]. The peptide-binding groove of the chicken MHC class I molecule contains α1 and α2 domains [18], so only those two domains were used for modelling and analysis in this study. BLASTP search results showed that the chicken MHC class I molecule 3BEV (PDB accession number) belonging to the B21 haplotype had the highest sequences identity and similarity with the four target sequences. The sequence identity and similarity between 3BEV and B4, B12, B15, and B19 were 84.3%, 84.3%, 87.1%, and 89.9%, and 89.3%, 89.9%, 91%, and 93.3%, respectively. The sequence alignments of the four target sequences and 3BEV are shown in Fig. 1. 3BEV has been crystallized to a high resolution of 2.1 Å, so it was selected as the template for modelling the four protein structures.

**Figure 2:**
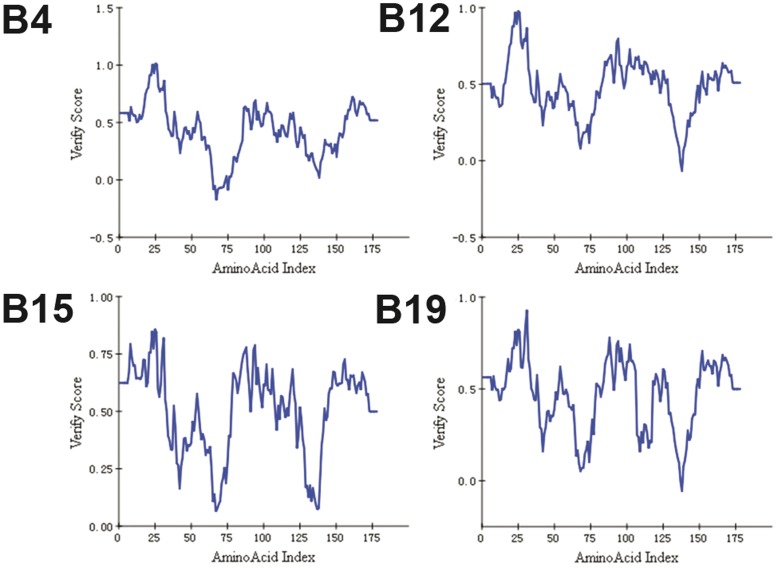
Verification score plots of the models (plots generated using DS2.5). The verification scores of all residues in B15 were greater than zero, whereas one residue in B12 and B19 and three residues in B4 were less than zero.

**Figure 3:**
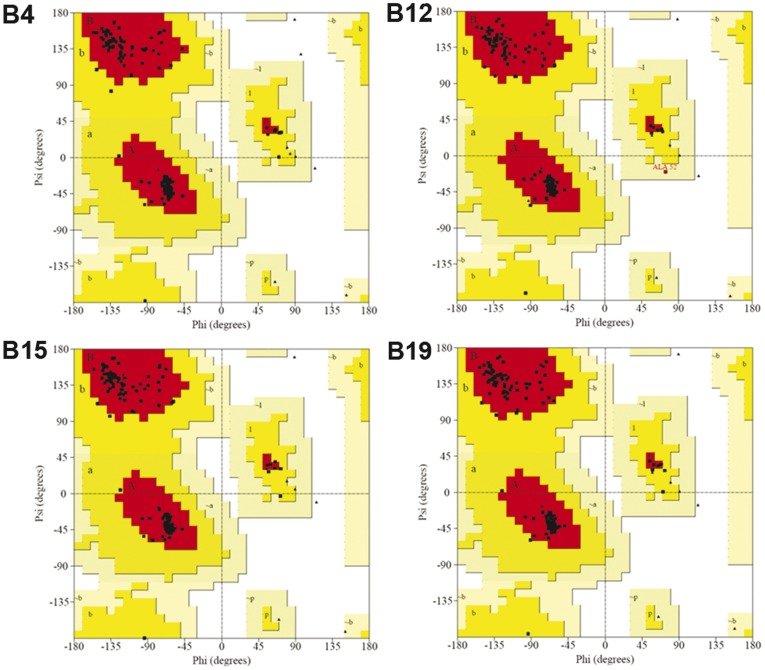
Ramachandran plots of the models (B4, B12, B15, and B19) produced using PROCHECK. The most favoured regions are coloured in red. Allowed, generously allowed, and disallowed regions are indicated as yellow, light yellow, and white regions, respectively.

The initial crude homology models were built using MODELLER and refined using modules for loop refinement and CHARMM minimization in DS2.5. Thus, the final structures were of high quality. The Profiles-3D scores of all amino acid residues in the four structures were greater than zero (Fig. 2), while an evaluation of the stereochemical quality of the models using PROCHECK showed that no residues were in the disallowed regions of the Ramachandran plots (Fig. 3). This indicated that the backbone dihedral angles, phi and psi, of the four structure models were reasonably accurate.

### Structural Differences

**Figure 4:**
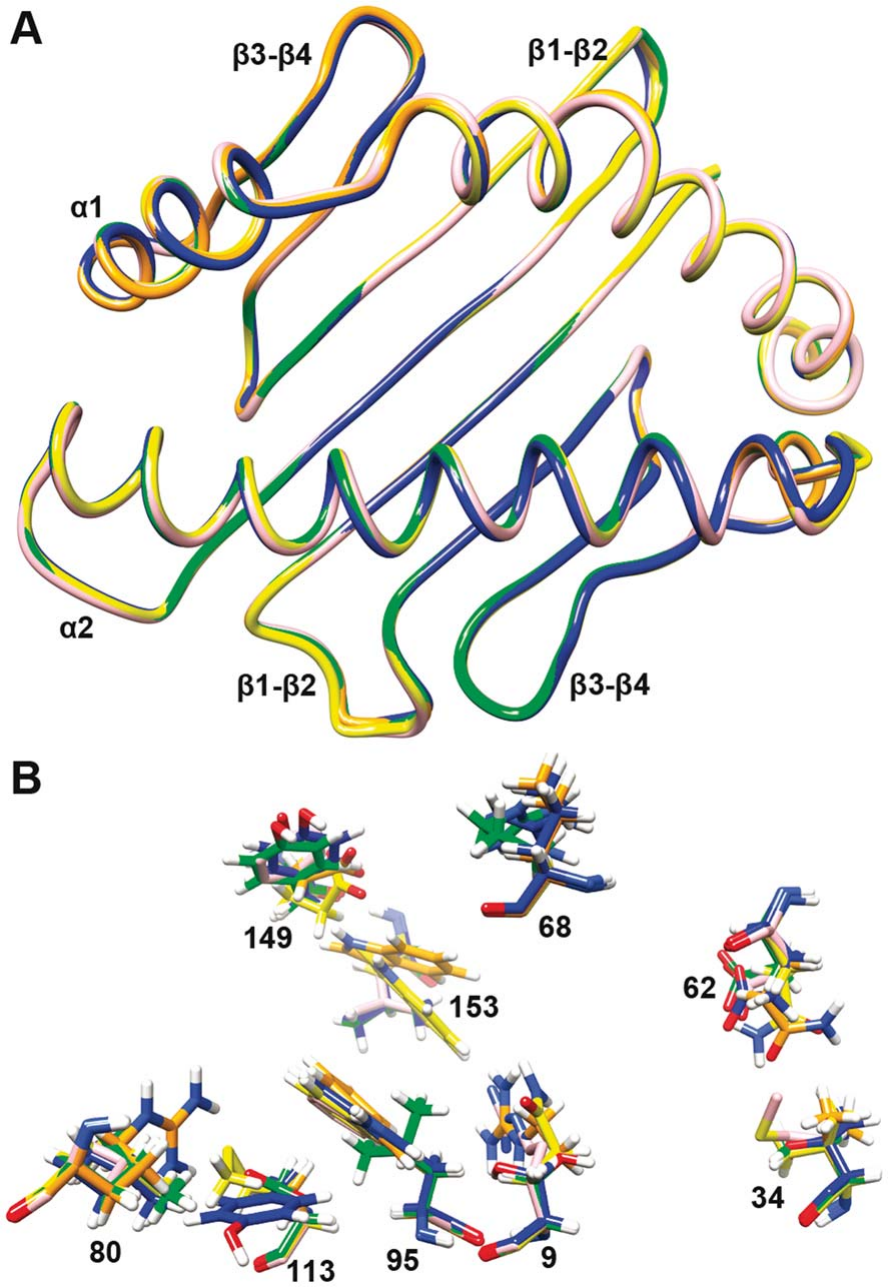
Superimposition of the homology modelling models (B4 in orange, B12 in yellow, B15 in green, and B19 in blue, with the template B21 in pink). (A) The structures are shown as Cα wires. The peptide-binding groove of each model contains α1 and α2 domains, where each domain is composed of one α-helix and four β-sheets. (B) The key residue differences are displayed as sticks in the corresponding region.

The four models are similar to the previously reported B21 haplotype. The peptide-binding domain is formed by two helices at the top and an eight-stranded sheet at the bottom (Fig. 4A). The pairwise root mean-square differences (RMSD) between the Cα positions of 3BEV and the structures of the B4, B12, B15, and B19 haplotypes are very small (0.23, 0.20, 0.24, and 0.30 respectively). However, there are some variations around the peptide-binding grooves and these differences may determine differences in their peptide-binding properties. Specific residues in the binding groove have an essential role in peptide binding. Figure 4B shows the key residues comprising the binding site, when all the models and the template were superimposed.

**Figure 5:**
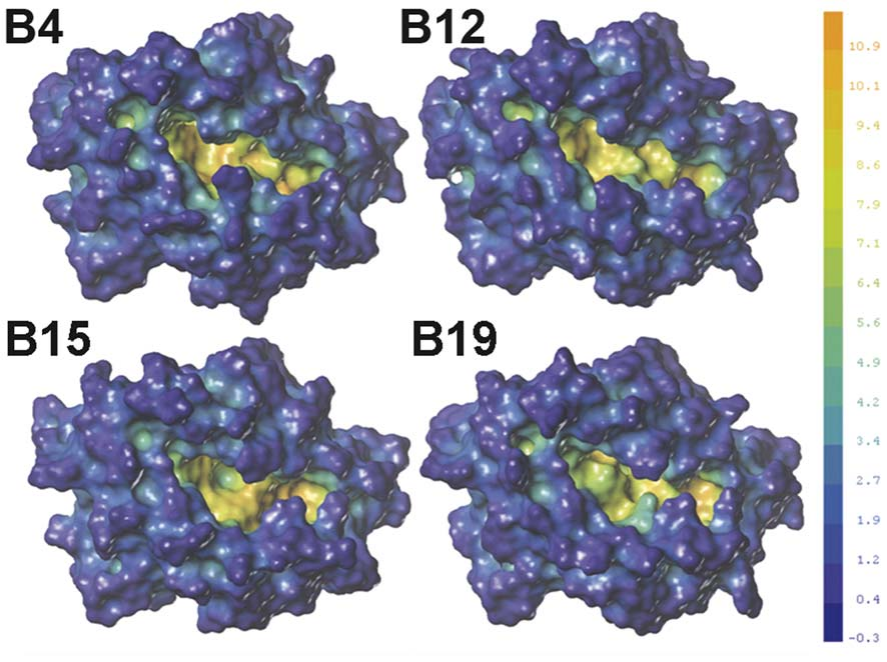
Comparison of the cavity depth and volume in the models. The deep blue colour represents the outermost surface of the structure, whereas the orange colour represents the deepest part of the cavity. All surfaces were rendered at the same scale. The cavity volumes of structures are 662.45 Å^3^ (B4), 637.677 Å^3^ (B12), 582.595 Å^3^ (B15), and 581.676 Å^3^ (B19). The maps and data were generated using SYBYL 8.1.

Peptide binding to a given class I MHC molecule requires the presence of anchor residues that complement the physicochemical characteristics of the specificity pockets [36]. Anchor residues are usually found at peptide position 2 (P2), where they interact with pocket B, but sometimes at position 5 or 6 (P5 or P6), where they interact with pocket C or E. A C-terminal residue (PC) also typically binds in pocket F [8]. The anchor residues Glu or Asp (P2) in B4 have an electrostatically favourable interaction with the side-chain of the pocket B residue Aspα9, which points up from the β-sheet. Similarly, anchor residues P2 Arg in B15 and B19 provide an electrostatically favourable interaction with the negatively-charged residues Aspα34 and Gluα62, the side-chains of which point towards the binding groove from the α-helix. The presence of Glu8 or Asp8 as major residues in the C-terminal position of the B4 motif is unprecedented, and there are no previous mammalian examples of negatively-charged residues in the C-terminal anchor position [16]. It is possible that the positively-charged Argα80 residue of the F pocket is the key to the binding of anchor residues P8Glu or P8Asp. There is an anchor residue in the central cavity region corresponding to pocket C in B12 and B4. This is very similar to the mammalian MHC class I molecules H-2 Kb and H-2 Db, and it may be related to the Glyα68(69) residue without a side-chain located in the α-helix (the residue number in parentheses is derived from mammals, whereas that without parentheses is from the chicken). The volumes of the binding grooves in B4 and B12 are 662.45 Å^3^ and 637.677 Å^3^, respectively, which are greater than those of B15 (582.595 Å^3^) and B19 (581.676 Å^3^) (Fig. 5). This may explain why the former have anchor residues in the middle region of their binding peptides. In general, anchor residues point downwards into the binding groove, which allows them to fill the pocket and maintain the stability of the peptide-MHC complex.

**Figure 6:**
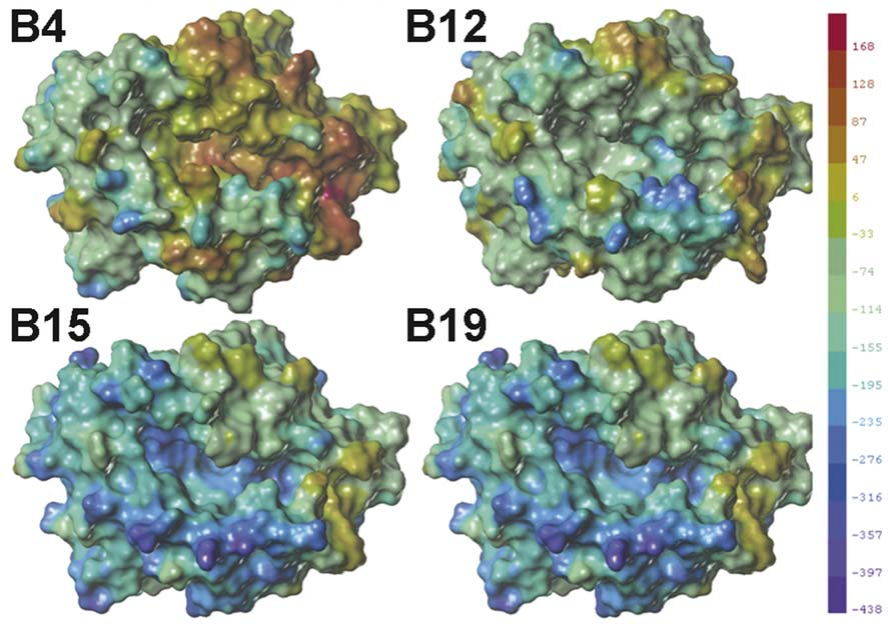
Comparison of molecular electrostatic potential (EP) surfaces of the models. The deep blue colour represents the most negative potential, whereas the deep red colour represents the most positive potential. In order to make valid comparisons among the structures, the electrostatic potentials are shown at the same scale. The maps and data were generated using SYBYL 8.1.

**Figure 7:**
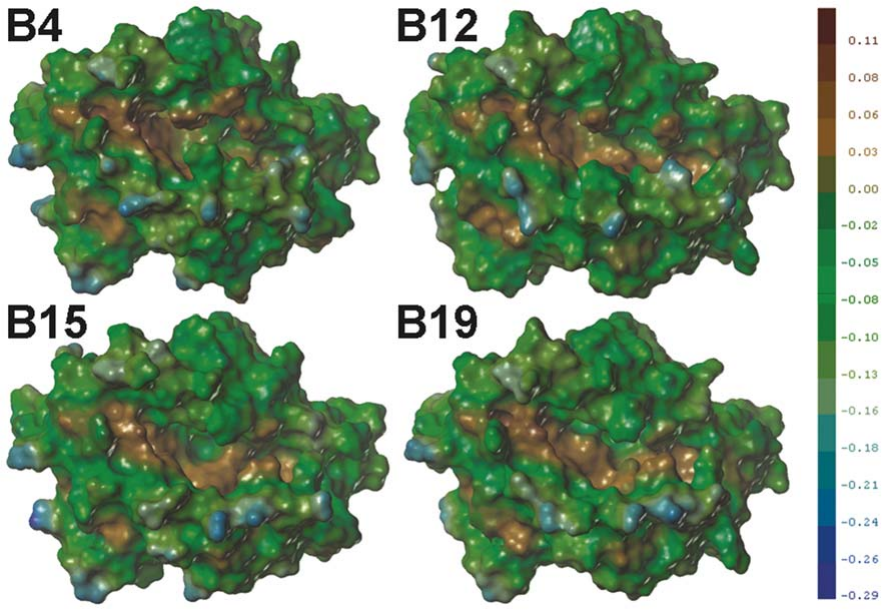
Comparison of molecular lipophilic potential (LP) of the surfaces of the models. The deep blue colour represents the most hydrophilic parts of the surface, whereas the deep brown colour represents the most lipophilic parts of the surface. All surfaces were rendered at the same scale shown in the legend. The maps and data were generated with SYBYL 8.1.

**Figure 8:**
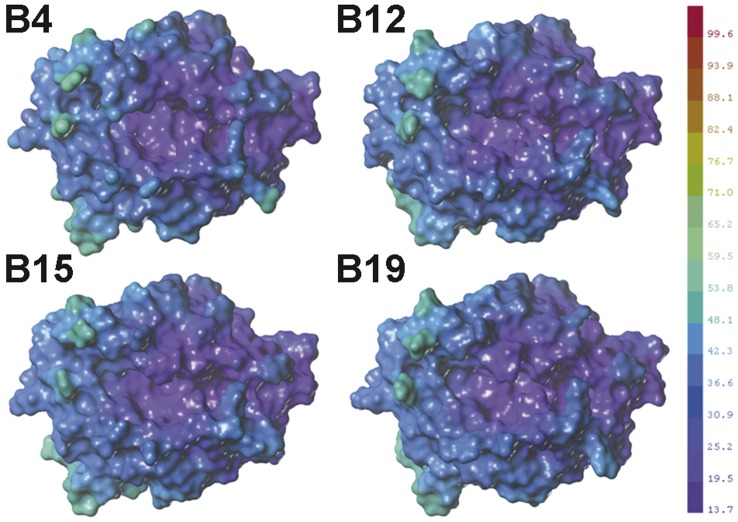
Comparison of the molecular flexibility (FX) of the models. The purple colour represents the most flexible parts of the structures, whereas the deep red colour represents the most rigid parts of the structure. All surfaces were generated at the same scale shown in the legend. The maps and data were generated using SYBYL 8.1.

The electrostatic potential of the peptide-binding groove of the B4 haplotype is highly positively-charged, whereas those of the B15 and B19 haplotypes are negatively-charged and that of B12 is neutral (Fig. 6). The peptide-binding grooves are highly lipophilic in all models (Fig. 7). The B pockets of the peptide-binding grooves are more flexible than the F pockets in all structures (Fig. 8), which suggests that the variation of the anchor residues in the B pocket is greater than in the F pocket.

### Surflex-Dock was Appropriate for Docking Peptides with MHC Class I Molecules

As described in the Materials and Methods, 50 peptide-MHC complexes were selected from the PDB database to validate the docking process. All the peptides were extracted from the complexes, before they were re-docked to the corresponding MHC molecules using Surflex-Dock. When compared with the corresponding initial complexes, the average RMSD was 1.8 and the docking scores were all greater than 8.0 (Table 2). Docking was also successfully performed with v-src C-tail peptide_517–524_ (LPACVLEV) and an identified T cell epitope [23] to the modelled B12 MHC class I molecule, where the docking score was 10.91 and the docking complex conformation was rational. Thus, it was appropriate to use Surflex-Dock for screening T cell epitopes based on their homology-modelled structures.

**Table 2:** Docking results for 50 peptide-MHC complexes using Surflex-Dock.

PDB	Score	RMSD(Å)	MHC-I
1I4F	17.03	2.875	HLA-A*0201
1HOC	18.29	1.486	H-2D^b^
1G7P	19.59	1.403	H-2K^b^
1FZJ	13.55	2.651	H-2K^b^
1AKJ	23.95	1.883	HLA-A*0201
1A1O	15.81	1.779	HLA-B*5301
1A1M	18.06	1.587	HLA-B*5301
1A1N	15.47	1.272	HLA-B*3501
1BQH	15.55	2.849	H-2K^b^
1EEZ	18.46	1.902	HLA-A*0201
1EEY	17.61	1.684	HLA-A*0201
1E27	19.93	2.609	HLA-B*5101
1FG2	18.85	1.889	H-2D^b^
1FZK	13.08	1.151	H-2K^b^
1HHH	23.29	1.459	HLA-A*0201
1HHK	19.55	0.914	HLA-A*0201
1HHG	20.63	2.057	HLA-A*0201
1LK2	19.73	1.036	H-2K^b^
1NAM	21.5	1.108	H-2K^b^
1MO5	25	0.796	HLA-B*0801
1Q94	18.27	1.447	HLA-A*1101
1RKO	15.42	0.892	H-2K^b^
1UXW	24.99	1.63	HLA-B*2709
1QEW	21.86	1.195	HLA-B*3501
1WBY	17.87	2.596	H-2D^b^
1WBZ	15.08	1.102	H-2K^b^
1XR8	21.73	2.327	HLA-B*1501
1X7Q	23.08	1.349	HLA-A*1101
1JPG	24.14	1.704	H-2D^b^
1JPF	21.04	2.257	H-2D^b^
1QO3	23.77	2.358	H-2D^b^
2BSR	19.76	2.216	HLA-B*2705
2BSS	22.14	2.733	HLA-B*2705
2BST	20.79	2.139	HLA-B*2705
2BVO	17.25	2.925	HLA-B*5703
2BVP	22.04	1.394	HLA-B*5703
2BVQ	16.01	1.094	HLA-B*5703
2C7U	17.31	1.621	HLA-A*0201
2FWO	20.95	1.871	H-2K^d^
2GUO	13.68	1.334	HLA-A*0201
2HJK	11.96	2.028	HLA-B*5703
2VAA	21	1.302	H-2K^b^
2VAB	14.89	1.439	H-2K^b^
2VLJ	17.55	1.185	HLA-A*0201
2V2X	18.17	1.659	HLA-A*0201
3BUY	21.18	1.887	H-2D^b^
3CPL	18.86	1.25	H-2D^b^
3DX6	21.68	2.542	HLA-B*4402
3D25	16.94	2.409	HLA-A*0201
3GIV	19.45	1.583	HLA-A*0201

### Peptides Predicted as Potential T Cell Epitopes in AIV H5 NP in the Chicken

Based on the assessment results, the criteria for T cell epitope prediction using Surflex-Dock were defined as follows: a) the peptide made close contact with the groove and it docked in the correct direction; b) the anchor residues bound to the anchor site in a rational conformation; and c) the docking score of the complex was greater than 8.0. A total of 25 potential T cell epitope peptides were predicted (eight for B4, six for B12, two for B15, and seven for B19). Nine peptides, which marked in bold in table 1, were selected to test their T cell responses in vitro. The binding energies of those complexes were also calculated. The results are shown in Table 1.

### Immune Response Successfully Induced by NP DNA Vaccine Inoculation in Chickens

**Figure 9:**
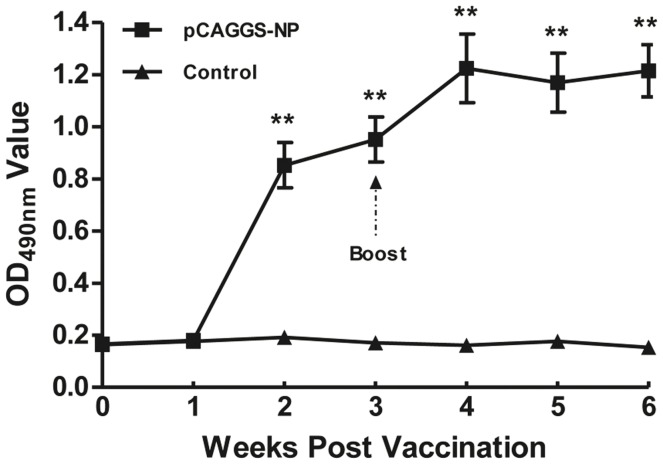
NP antibody response in chickens vaccinated with plasmid pCAGGS-NP, as detected using ELISA. A significant NP antibody increase was detected two weeks post-immunization in chickens immunized with pCAGGS-NP compared to chickens in control group, which increased further after the boost and remained at a high level until the end of the experiment.

Peptide stimulation experiments were conducted using splenic lymphocytes derived from NP DNA vaccine-immunized chickens to verify some of the predicted potential T cell epitopes. To assess the immune effects of the vaccine, serum NP antibody was detected using the ELISA method. Compared to the control group, a significant increase (*P*<0.01) in blood NP antibody was observed in the immunized group two weeks after the first vaccination. After the boost, the blood NP antibody level of the immunized group increased further and it remained at a high level throughout the duration of the experiment (Fig. 9). This indicated that the immune systems of the chickens were activated by the vaccine immunization and that the splenic lymphocytes of chickens were sensitized.

### Activation of Lymphocytes Using Peptides NP_89–9_ and NP_198–206_

**Figure 10:**
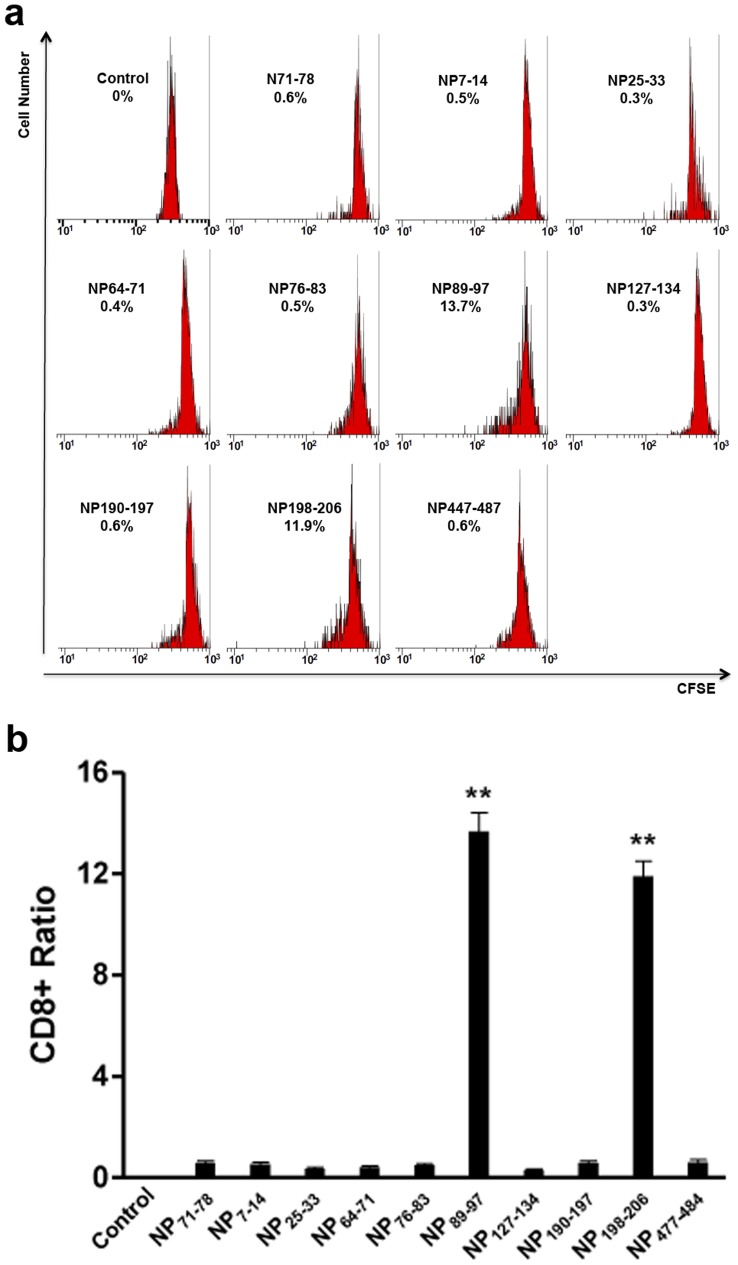
CD8^+^ T cell proliferation after stimulation with different peptides. (a) Flow cytometry results. The control was normal spleen lymphocytes. The N_71–78_ group was spleen lymphocytes stimulated with the irrelevant peptide N_71–78_, whereas the remainder were spleen lymphocytes stimulated with peptides derived from NP. CD8^+^ T cell proliferation was increased by 13.7% and 11.9% in cells stimulated with NP_89–97_ and NP_198–206_, respectively. CD8^+^ T cells were labelled using CESF and detected by Cytomics FC500 MCL(Beckman). (b) Statistics of flow cytometry results. CD8^+^ T cell proliferation in cells stimulated with NP_89–97_ and NP_198–206_ was significant higher than cells stimulated with unrelated peptide N_71–78_.

**Figure 11:**
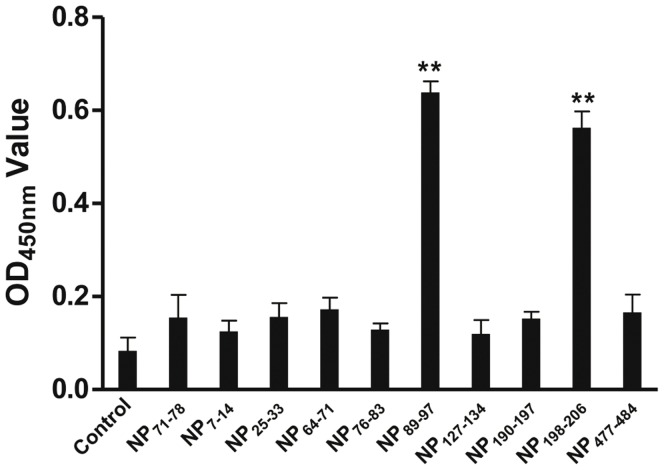
Secretion of chicken IFN-γ in cells stimulated with peptides, as detected by ELISA. The control was spleen cells that received no peptide stimulation. N_71–78_ were spleen lymphocytes stimulated with the irrelevant peptide N_71–78_, whereas the remainder were spleen lymphocytes stimulated with peptides derived from NP. Significant increases of IFN-γ were observed in cells stimulated with NP_89–97_ and NP_198–206_ when compared to cells stimulated with unrelated peptide N_71–78_.

To verify the predicted T cell epitopes in NP, ten synthetic peptides (Top nine of the 25 predicted peptides from NP and one unrelated peptide) were incubated with sensitized splenic lymphocytes for 5 d. Flow cytometry analysis showed that the proliferation of CD8^+^ T lymphocytes increased by 13.7% and 11.9% in cells stimulated with the peptides NP_89–97_ and NP_198–206,_ respectively (Fig. 10). Chicken IFN-γ concentration in cells stimulated using peptides NP_89–97_ and NP_198–206_ were significantly higher than the control and unrelated peptide-stimulated cells (Fig. 11). These results demonstrate that the peptides NP_89–97_ and NP_198–206_ are NP T cell epitopes in chickens of certain haplotypes.

## Discussion

The objective of this study was to predict and verify T cell epitopes in the H5N1 AIV NP in chicken. Using a motif combined with a structure-based method, 25 potential T cell epitope peptides were predicted in the H5N1 AIV NP in chickens of B4, B12, B15, and B19 haplotypes. NP_89–97_ and NP_198–206_ were found to induce a significant proliferation of CD8^+^ T lymphocytes and they increased the secretion of chicken IFN-γ in sensitized splenic lymphocytes. These data suggest that peptides NP_89–97_ and NP_198–206_ are NP T cell epitopes in chickens of certain haplotypes. This study is important for the following two reasons. First, this is the first study to determine the structural characteristics of the peptide-binding domains of chicken MHC class I molecules belonging to the B4, B12, B15, and B19 haplotypes using a combined motif-structure method to predict T cell epitopes in chickens. Second, NP_89–97_ and NP_198–206_ are the first two T cell epitopes to be identified in AIV NP in chickens of certain haplotypes.

Homology modelling is widely used in many areas of structure-based analysis and study [37], [38]. However, there are few studies of chicken MHC class I molecules compared with those of human or mouse. The only chicken MHC class I molecule structure that has been solved is the B21 haplotype [18]. Thus, there is little information available on the structure and function of chicken MHC class I molecules. Therefore, the current study used homology modelling to investigate the peptide-binding domains of chicken MHC class I molecules belonging to the B4, B12, B15 and B19 haplotypes. To the best of our knowledge, this is the first attempt to understand the peptide-binding properties of these molecules based on their structures. The assessment indicated that the models were of high quality in terms of their folding and they were suitable for structure-based T cell epitope prediction. The structural characteristics of the peptide-binding properties of these MHC class I molecules were described in the results section. Only four haplotypes with known motifs were selected in this study. However, it is possible to use this solution to predict T cell epitopes of MHC class I molecules belonging to different haplotypes for other antigens in chickens, because the only difference would be the increased number of peptides required for molecular docking.

This study found that 25 out of 75 peptides were potential T cell epitopes in the H5 AIV NP in chickens of the four haplotypes. An analysis of previous results showed that some of those peptides have been identified as T cell epitopes in humans. There is evidence that NP_91–99_ (KTGGPIYKR), NP_361–375_ (RGVQIASNENMETME), and NP_174–184_ (RRSGAAGAAVK), which are derived from the H3N2, H3N2, and H1N1, respectively, are T cell epitopes, with MHC restriction alleles of HLA-A68, H-2Db, and HLA-B27, respectively [39]. Of these peptides, NP_89–97_ (PKKTGGPIY) and NP_362–369_ (GVQIASNE) are completely conserved in all influenza virus strains, while NP_179–186_ (AGAAVKGV) is relatively conserved. Some potential epitopes were also shared by several haplotypes. Chickens used for meat and egg production are always heterozygotes, so the identification of these shared T cell epitopes will facilitate the development of broad-spectrum protective vaccines for chickens. Furthermore, epitopes shared by birds and humans are of great importance in the design of rational vaccines for protecting humans and birds from AIV infection.

This study used a DNA vaccine plasmid expressing the NP from A/Goose/Gong Dong/1/96 (H5N1). A DNA vaccine expressing HA from A/Goose/Gong Dong/1/96 (H5N1) has been found to elicit antibody responses and protect chickens against challenge with HPAI virus [25]. Methods used for producing the HA-expression DNA vaccine were adopted when preparing the DNA expression plasmid pCAGG-NP. NP antibody responses were detected in immunized chickens, suggesting that this vaccine elicits successful immune responses to the NP antigen in chickens. The T cell responses were not detected directly in this study, but it was inferred that a successful antibody response would have been accompanied by a successful T cell response based on our knowledge of the DNA vaccine.

Nine of the peptides were synthesized and used to stimulate sensitized splenic lymphocytes to verify that they were epitopes. Increases in the proliferation of CD8^+^ T lymphocytes and the secretion of chicken IFN-γ demonstrated the antigenicity of these peptides. NP_89–97_ (PKKTGGPIY) and NP_198–206_ (KRGINDRNF) induced significant T cell responses in splenic lymphocytes. An analysis of the prediction results showed that NP_89–97_ was predicted to be a T cell epitope for both B15 and B19, while NP_198–206_ also belonged to the B19 haplotype. Thus, it is suggested that the major haplotype of the experimental SPF chickens might be B19. NP_89–97_ also overlapped with a previously identified HLA-A68 restriction T cell epitope NP_91–99_ (KTGGPIYKR) of the H3N2 AIV [7]. This further verifies the significance of this epitope, which could be used in human and chicken vaccines to provide protection against different influenza virus subtypes.

Using in silico and in vitro approaches, this study identified two novel T cell epitopes NP_89–97_ (PKKTGGPIY) and NP_198–206_ (KRGINDRNF) in the H5N1 AIV NP in chickens of certain haplotypes. The method used in this investigation is applicable to predicting T cell epitopes for other antigens in chicken, while this study also extends our understanding of the mechanisms of the immune response to AIV in chickens.
